# Association between admission hemoglobin level and prognosis in sepsis patients based on a critical care database

**DOI:** 10.1038/s41598-024-55954-1

**Published:** 2024-03-03

**Authors:** Hongchun Peng, Yingjie Su, Ju Luo, Ning Ding

**Affiliations:** 1 Department of Orthopedics, The Affiliated Changsha Central Hospital, Hengyang Medical School, University of South China, Changsha, China; 2 Department of Emergency Medicine, The Affiliated Changsha Central Hospital, Hengyang Medical School, University of South China, No. 161 Shaoshan South Road, Changsha, 410004 Hunan China; 3 Department of Geriatrics, The Affiliated Changsha Central Hospital, Hengyang Medical School, University of South China, No. 161 Shaoshan South Road, Changsha, 410004 Hunan China

**Keywords:** Hemoglobin, Sepsis, Mortality, Anemia, Infectious diseases, Risk factors

## Abstract

This study aimed to explore the association between admission hemoglobin level and clinical outcomes in sepsis based on Medical Information Mart for Intensive Care IV(MIMIC-IV) database. It was a retrospective study. Sepsis patients in the database were included. Data extraction from MIMIC-IV was performed by PostgreSQL 9.6 software. Three different models including crude model (adjusted for none), model I (adjusted for gender and age) and model II (adjusted for all potential cofounders) were constructed. A generalized liner model and a smooth fitting curve for indicating the relationship between hemoglobin level and 30-day mortality were performed. 6249 septic patients with a 30.18% of 30-day mortality were included. With 1 g/dl increment in hemoglobin level, the values of odds ratio (OR) in crude model, model I and model II were 0.96 (95% confidential interval (CI) 0.94–0.99, *P = *0.0023), 0.96 (95%CI 0.93–0.98, *P = *0.0010) and 0.87 (95%CI 0.79–0.95, *P = *0.0020), respectively. The smooth fitting curve indicated a non-linear relationship and the turning point was 7.2 g/dl. Compared the hemoglobin group *< *7.2 g/dl, the risk of 30-day mortality significantly decreased by 32% in the hemoglobin group ≥ 7.2 g/dl (OR = 0.68, 95%CI 0.51–0.93, *P = *0.0142). The non-linear relationship between admission hemoglobin level and 30-day mortality in sepsis was found. Hemoglobin supplementation might be beneficial for septic patients with hemoglobin level < 7.2 g/dl.

## Introduction

Hemoglobin, as an easily accessible lab finding, has been comprehensively applied for detecting individual status due to its various physiological functions^1^. Generally, hemoglobin is delicately regulated by genetical and environmental factors and differs from genders, ages, and life habits, while the levels of hemoglobin in healthy adults are usually comparatively stable^2^. As a main carrier of oxygen, hemoglobin level decreasing may result in oxygen deficiency in the body and lead to the hypoxic and ischemic injury of tissues and organs^3^. Previous researches have explored the relationships between hemoglobin and clinical outcomes in many disorders including cancer^4^, diabetes^5^, stroke^6^ and trauma^7^.

Sepsis, as dysregulated organ dysfunction due to infection, can develop anemia by decreased level of serum iron, insufficient production of erythropoietin and the damage of erythrocytes^8^. In turn, decreased levels of hemoglobin may aggravate organ dysfunction and bring about poorer prognosis^9^.

Since hemoglobin levels can impact on the development of sepsis, this research investigated the relationship between admission hemoglobin level and clinical outcomes in sepsis in a large public database. We aimed to provide some clinical evidences of hemoglobin supplementation in septic patients for doctors to do individualized treatment.

## Methods

### Database and data extraction

Our retrospective study utilized the data in Medical Information Mart for Intensive Care IV(MIMIC-IV) (https://mimic.mit.edu/iv/) database. MIMIC-IV database included all the patients admitted in intensive care unit(ICU) of the Beth Israel Deaconess Medical Center between 2008 and 2019^10,11^. Information including demographic data, laboratory variables, imaging findings and treatment records were comprehensively recorded in the database.

The website GitHub (https://github.com/MIT-LCP/mimic-iv) was applied for acquiring the codes for data extraction. Data extraction from MIMIC-IV was performed by PostgreSQL 9.6 software.

### Patients and variables

Septic patients in MIMIC-IV were included. Sepsis was diagnosed based on the definition of Sepsis 3.0^12^. Exclusion criteria were demonstrated as follow: (1) patients less than 18 years old; (2) without admission hemoglobin level; (3) patients with missing > 5% personal variables which were enrolled in our study.

Personal variables including age, gender, comorbidities (hypertension, diabetes, coronary artery disease (CAD) and renal disease) were extracted. The clinical outcomes including length of stay (LOS) in ICU and hospital, and 30-day mortality were included.

Vital signs and laboratory variables within 24 h after admission were also extracted as follow: systolic blood pressure (SBP), diastolic blood pressure (DBP), respiratory rate (RR), heart rate (HR), hemoglobin, red blood cells(RBC), hematocrit, white blood cells (WBC), platelet (PLT), creatinine, urea nitrogen, anion gap(AG), alanine aminotransferase (ALT), aspartate aminotransferase (AST), total bilirubin, total calcium, bicarbonate, prothrombin time (PT), thrombin time (TT), international normalized ratio(INR), lactate, chloride and sodium were extracted. The scores acute physiology and chronic health evaluation (APACHEII) and SOFA were also extracted. Only the first record of each variable in 24 h after admission was enrolled for analysis.

### Statistical analysis

Statistical analysis was performed by statistical software packages R (http://www.r-project.org) and EmpowerStats (http://www.empowerstats.com). Statistically significant was considered when a *P*-value less than 0.05.

Five groups based on quantiles of admission hemoglobin level (20% quantile, 40% quantile, 60% quantile, 80% quantile; Q0–Q4 groups) were constructed in the septic cohort: Q0(≤ 8.4 g/dl, n = 1189), Q1(8.5–9.6 g/dl, n = 1304), Q2(9.7–10.8 g/dl, n = 1190), Q3(10.9–12.1 g/dl, n = 1303), Q4(≥ 12.2 g/dl, n = 1263) (Table 1 and Supplementary Table 1).

**Table 1:** General variables of sepsis patients in the cohort.

Hemoglobin(g/dl) (quantiles)					
Variables	Total	Q0(≤ 8.4)	Q2(9.7–10.8)	Q4(≥ 12.2)	*P*-value
Number	6249	1189	1190	1263	
Age(years)	66.00 (55.00–77.00)	64.00 (54.00–74.00)	67.00 (56.00–77.00)	66.00 (55.00–77.00)	< 0.001
Gender (n,%)					< 0.001
Male	3541 (56.67%)	636 (53.49%)	624 (52.44%)	871 (68.96%)	
Female	2708 (43.33%)	553 (46.51%)	566 (47.56%)	392 (31.04%)	
Comorbidities (n,%)					
Hypertension	1302 (20.84%)	158 (13.29%)	263 (22.10%)	317 (25.10%)	< 0.001
Diabetes	203 (3.25%)	35 (2.94%)	38 (3.19%)	26 (2.06%)	0.015
CAD	570 (9.12%)	59 (4.96%)	123 (10.34%)	127 (10.06%)	< 0.001
Renal disease	271 (4.34%)	39 (3.28%)	70 (5.88%)	25 (1.98%)	< 0.001
SBP (mmHg)	110.00 (97.00–128.00)	109.00 (96.00–125.00)	110.00 (98.00–127.00)	112.00 (98.00–130.00)	0.012
DBP (mmHg)	63.00 (53.00–74.00)	60.00 (52.00–72.00)	62.00 (53.00–74.00)	66.00 (56.00–78.00)	< 0.001
HR (beats/min)	97.00 (83.00–112.00)	98.00 (84.00–113.00)	96.00 (82.00–112.00)	99.00 (84.00–114.00)	0.002
RR (beats/min)	21.00 (17.00–25.00)	21.00 (17.00–26.00)	20.00 (17.00–24.00)	21.00 (17.00–26.00)	0.048
Hemoglobin (g/dl)	10.20 (8.80–11.80)	7.60 (7.10–8.00)	10.20 (9.90–10.40)	13.30 (12.70–14.20)	< 0.001
RBC (*10^12^/l)	3.44 (2.94–3.98)	2.58 (2.32–2.82)	3.40 (3.20–3.64)	4.36 (4.08–4.72)	< 0.001
Hematocri t(%)	31.60 (27.20–36.20)	23.80 (22.10–25.30)	31.50 (30.20–32.80)	40.30 (38.30–43.40)	< 0.001
WBC (*10^9^/l)	12.50 (7.70–18.50)	11.10 (6.50–17.20)	12.30 (7.82–18.48)	13.20 (8.65–18.90)	< 0.001
PLT(*10^9^/l)	182.00 (117.00–264.00)	159.00 (84.00–266.00)	185.50 (123.00–269.00)	185.00 (129.00–248.00)	< 0.001
Creatinine (mg/dl)	1.40 (0.90–2.30)	1.40 (0.90–2.50)	1.30 (0.90–2.30)	1.30 (0.90–2.00)	0.003
Urea nitrogen (mg/dl)	28.00 (18.00–48.00)	32.00 (19.00–54.00)	29.00 (17.00–48.00)	26.00 (17.00–42.00)	< 0.001
AG (mmol/l)	16.00 (13.00–19.00)	15.00 (12.00–19.00)	16.00 (13.00–19.00)	17.00 (14.00–20.00)	< 0.001
ALT (IU/L)	29.00 (16.00–66.00)	25.00 (14.00–50.00)	29.00 (17.00–70.75)	36.00 (20.00–90.00)	< 0.001
AST (IU/L)	44.00 (24.00–99.00)	39.00 (22.00–85.00)	45.00 (25.00–101.75)	52.00 (28.00–126.00)	< 0.001
Total bilirubin (mg/dl)	0.80 (0.40–1.80)	0.70 (0.40–2.00)	0.70 (0.40–1.70)	0.90 (0.50–2.00)	< 0.001
Total calcium (mg/dl)	8.00 (7.50–8.60)	7.90 (7.30–8.50)	8.00 (7.43–8.50)	8.20 (7.60–8.80)	< 0.001
Bicarbonate (mmol/l)	21.00 (18.00–25.00)	21.00 (18.00–24.00)	21.00 (18.00–24.00)	21.00 (18.00–25.00)	0.113
INR	1.40 (1.20–1.90)	1.50 (1.30–2.10)	1.40 (1.20–1.80)	1.40 (1.20–1.80)	< 0.001
PT(s)	15.40 (13.30–20.10)	16.30 (13.90–22.10)	15.30 (13.30–19.78)	14.90 (12.80–19.30)	< 0.001
TT(s)	33.00 (28.70–40.70)	34.20 (29.40–42.60)	32.80 (28.70–40.10)	32.70 (28.30–39.85)	< 0.001
Lactate (mmol/l)	2.00 (1.40–3.10)	1.80 (1.20–2.90)	2.00 (1.40–3.10)	2.40 (1.60–3.60)	< 0.001
Chloride (mmol/l)	103.00 (98.00–107.00)	103.00 (98.00–108.00)	103.00 (98.00–108.00)	103.00 (98.00–107.00)	0.580
Sodium (mmol/l)	138.00 (134.00–141.00)	137.00 (134.00–141.00)	138.00 (134.00–141.00)	138.00 (135.00–141.00)	0.006
SOFA	3.00 (2.00–5.00)	3.00 (2.00–5.00)	3.00 (2.00–4.00)	2.00 (2.00–4.00)	< 0.001
APACHEII	12.00 (9.00–15.00)	13.00 (11.00–16.00)	11.00 (9.00–14.00)	11.00 (9.00–14.00)	< 0.001
Outcomes					
LOS in ICU (days)	4.75 (2.17–10.60)	4.55 (2.13–10.31)	4.55 (2.16–10.51)	5.27 (2.21–11.83)	0.261
LOS in hospital (days)	11.63 (6.39–20.84)	12.28 (6.76–22.43)	11.59 (6.49–19.58)	11.71 (5.93–21.45)	0.115
30-day mortality (n,%)	1886 (30.18%)	407 (34.23%)	362 (30.42%)	352 (27.87%)	0.001

ALT, alanine aminotransferase; AST, aspartate aminotransferase; CAD, coronary artery disease; SBP, systolic blood pressure; DBP, diastolic blood pressure; HR, heart rate; RR, respiratory rate; WBC, white blood cells; PLT, platelet; RBC, red blood cells; PT, prothrombin time; TT, thrombin time; INR, international normalized ratio; AG, anion gap; SOFA, sequential organ failure assessment; APACHE, acute physiology and chronic health evaluation; LOS, length of stay; ICU, intensive care unit; IQR, interquartile ranges.

In this research, continuous parameters were demonstrated as medians and/or interquartile ranges (IQR). Categorial parameters were demonstrated as percentages and/or frequencies. First, Chi-squared test and Kruskal–Wallis test were utilized to compare the variables between five different groups. Second, investigating the relationships between different variables and 30-day mortality was performed by univariate analysis. Third, investigating the relationship between admission hemoglobin level and 30-day mortality was implemented by three different models including crude model (adjusted for none), model I (adjusted for gender and age) and model II (adjusted for all potential cofounders). The odds ratio (OR) of outcomes and the 95% confidence interval (95% CI) for each g/dL change in hemoglobin level were calculated. Fourth, hemoglobin was changed from continuous variable into categorical variable (quantiles, Q0–Q4), and the associations between hemoglobin (quantiles) and three models were also explored. The *P* values for trend of categorized hemoglobin level in all models were confirmed. Fifth, a smooth fitting curve by the generalized linear model for indicating the relationship between hemoglobin level and 30-day mortality after adjusted for all potential cofounders were performed. Standard linear model and two-piecewise linear model were applied to examine which model was the better one for fitting the relationship between hemoglobin level and 30-day mortality. The best fitting model was confirmed on the basis of the *P*-value of the log-likelihood ratio test. If the *P*-value < 0.05, the two-piecewise linear model was better. If the *P*-value ≥ 0.05, the standard linear model was better. Sixth, Kaplan–Meier analysis was applied to analyze and compare for cumulative hazard of 30-day mortality between five groups. At last, we did the subgroups analyses by stratified models for discussing the stability of our results in subgroups.

## Ethical approval and consent to participate

This study was performed in accordance with the Helsinki Declaration of 1964 and its later amendments. MIMIC-IV was an anonymized public database. To apply for access to the database, we passed the Protecting Human Research Participants exam (No.32900964). The project was approved by the institutional review boards of the Massachusetts Institute of Technology (MIT) and Beth Israel Deaconess Medical Center (BIDMC) and was given a waiver of informed consent.

## Results

### General variables of sepsis patients in the cohort

A total of 6249 patients were included in our study (Supplementary Fig. 1). In Table 1, general characteristics of the cohort were demonstrated. The 30-day mortality was 30.18% (n = 1886). The median age was 66 years old and males accounted for 56.67% (n = 3541). Hypertension was the most frequency comorbidity (n = 1302). The median level of hemoglobin was 10.20 g/dl. The scores of SOFA and APACHEII were 3.00 (2.00–5.00) and 12.00 (9.00–15.00), respectively. The days of LOS in ICU and hospital were 4.75 (2.17–10.60) and 11.63 (6.39–20.84), respectively. Compared different variables in five groups based on quantiles of hemoglobin level, three groups Q0, Q2 and Q4 groups were illuminated in Table 1. No significant differences were showed in the variables including bicarbonate (*P = *0.113), chloride (*P = *0.580), LOS in ICU (*P = *0.261) and LOS in hospital (*P = *0.115). 30-day mortality showed significant difference (*P = *0.001) and the mortalities in Q0 group and Q4 group were 34.23% and 27.87%, respectively.

### Univariate analysis for 30-day mortality in sepsis

Table 2 indicated univariate analysis for 30-day mortality in sepsis. Variables including age (*P < *0.0001), diabetes(*P = *0.0274), CAD (*P = *0.0450), renal disease(*P = *0.0043), SBP (*P = *0.0018), DBP(*P = *0.0388), RR(*P = *0.0004), hemoglobin(*P = *0.0023), RBC(*P < *0.0001), PLT (*P < *0.0001), creatinine (*P < *0.0001), urea nitrogen (*P < *0.0001), AG (*P < *0.0001), ALT (*P < *0.0001), AST (*P < *0.0001), total bilirubin (*P < *0.0001), bicarbonate (*P < *0.0001), INR (*P < *0.0001), PT (*P < *0.0001), TT (*P < *0.0001), lactate (*P < *0.0001), chloride (*P < *0.0001), sodium (*P = *0.0053), SOFA (*P < *0.0001) and APAHCEII (*P < *0.0001) were associated with 30-day mortality in sepsis.

**Table 2:** Univariate analysis for 30-day mortality in sepsis.

Variables	Univariate (OR,95%CI, P)
Age(years)	1.02 (1.02, 1.02) < 0.0001
Gender	
Female	Ref
Male	1.02 (0.92, 1.14) 0.6684
Hypertension	
No	Ref
Yes	0.95 (0.83, 1.09) 0.4573
Diabetes	
No	Ref
Yes	0.69 (0.50, 0.96) 0.0274
CAD	
No	Ref
Yes	1.21 (1.00, 1.45) 0.0450
Renal disease	
No	Ref
Yes	1.44 (1.12, 1.85) 0.0043
SBP (mmHg)	1.00 (0.99, 1.00) 0.0018
DBP (mmHg)	1.00 (0.99, 1.00) 0.0388
HR (beats/min)	1.00 (1.00, 1.00) 0.0678
RR (beats/min)	1.01 (1.01, 1.02) 0.0004
Hemoglobin (g/dl)	0.96 (0.94, 0.99) 0.0023
RBC (*10^12^/l)	0.83 (0.77, 0.89) < 0.0001
Hematocrit (%)	0.99 (0.99, 1.00) 0.1104
WBC (*10^9^/l)	1.00 (1.00, 1.01) 0.3414
PLT (*10^9^/l)	1.00 (1.00, 1.00) < 0.0001
Creatinine (mg/dl)	1.07 (1.04, 1.10) < 0.0001
Urea nitrogen (mg/dl)	1.01 (1.01, 1.01) < 0.0001
AG (mmol/l)	1.06 (1.05, 1.07) < 0.0001
ALT (IU/L)	1.00 (1.00, 1.00) < 0.0001
AST (IU/L)	1.00 (1.00, 1.00) < 0.0001
Total bilirubin (mg/dl)	1.06 (1.04, 1.07) < 0.0001
Total calcium (mg/dl)	1.04 (0.98, 1.10) 0.1689
Bicarbonate (mmol/l)	0.98 (0.97, 0.99) < 0.0001
INR	1.21 (1.16, 1.26) < 0.0001
PT(s)	1.02 (1.01, 1.02) < 0.0001
TT(s)	1.01 (1.01, 1.01) < 0.0001
Lactate( mmol/l)	1.22 (1.19, 1.25) < 0.0001
Chloride (mmol/l)	0.98 (0.97, 0.99) < 0.0001
Sodium (mmol/l)	0.99 (0.98, 1.00) 0.0053
SOFA	1.22 (1.19, 1.26) < 0.0001
APACHEII	1.10 (1.09, 1.12) < 0.0001

ALT, alanine aminotransferase; AST, aspartate aminotransferase; CAD, coronary artery disease; SBP, systolic blood pressure; DBP, diastolic blood pressure; HR, heart rate; RR, respiratory rate; WBC, white blood cells; PLT, platelet; RBC, red blood cells; PT, prothrombin time; TT, thrombin time; INR, international normalized ratio; AG, anion gap; SOFA, sequential organ failure assessment; APACHE, acute physiology and chronic health evaluation; OR, odds ratio; CI, confidential interval.

### Association of admission hemoglobin level with 30-day mortality in three models

In Table 3, relationships between hemoglobin level and 30-day mortality were explored in three models. With 1 g/dl increment in hemoglobin level, the values of OR in crude model (adjusted for none), model I(adjusted for age and gender) and model II(adjusted for all potential cofounders) were 0.96 (95%CI 0.94–0.99, *P = *0.0023), 0.96 (95%CI 0.93–0.98, *P = *0.0010) and 0.87 (95%CI 0.79–0.95, *P = *0.0020), respectively. Moreover, we changed the hemoglobin level from continuous variable into categorial variables (quantiles) and also investigated the relationships in three models. In all three models, Q4 group had the lowest risk of 30-day mortality (crude model: OR = 0.74, 95%CI 0.63–0.88, *P = *0.0007; model I: OR = 0.72, 95%CI 0.60–0.86, *P < *0.0001; model II: OR = 0.42, 95%CI 0.28–0.64, *P < *0.0001).

**Table 3:** Association of admission hemoglobin level with 30-day mortality in three models.

OR(95%CI), *P* value			
Exposure	Crude model	Model I	Model II
Hemoglobin level(per 1 g/dl increment)	0.96 (0.94, 0.99) 0.0023	0.96 (0.93, 0.98) 0.0010	0.87 (0.79, 0.95) 0.0020
Hemoglobin level(g/dl) quantiles			
Q0(≤ 8.4 g/dl, n = 1189)	Ref	Ref	Ref
Q1(8.5–9.6 g/dl, n = 1304)	0.87 (0.74, 1.03) 0.1179	0.84 (0.71, 1.00) 0.0470	0.81 (0.66, 1.00) 0.0469
Q2(9.7–10.8 g/dl, n = 1190)	0.84 (0.71, 1.00) 0.0470	0.80 (0.67, 0.95) 0.0129	0.71 (0.55, 0.91) 0.0074
Q3(10.9–12.1 g/dl, n = 1303)	0.73 (0.61, 0.86) 0.0002	0.68 (0.57, 0.81) < 0.0001	0.55 (0.40, 0.75) 0.0001
Q4(≥ 12.2 g/dl, n = 1263)	0.74 (0.63, 0.88) 0.0007	0.72 (0.60, 0.86) 0.0002	0.42 (0.28, 0.64) < 0.0001
P for trend	< 0.0001	< 0.0001	< 0.0001

Crude model adjusted for: None; Model I adjusted for: age; gender; Model II adjusted for: age; gender; HR; SBP; DBP; RR; AG; ALT, AST, bicarbonate, INR, total bilirubin; total calcium; creatinine; chloride; hematocrit; lactate; PLT; PT; TT; RBC; urea nitrogen; WBC; sodium; renal disease; CAD; diabetes; hypertension; SOFA; APAHCEII.

ALT, alanine aminotransferase; AST, aspartate aminotransferase; CAD, coronary artery disease; SBP, systolic blood pressure; DBP, diastolic blood pressure; HR, heart rate; RR, respiratory rate; WBC, white blood cells; PLT, platelet; RBC, red blood cells; PT, prothrombin time; TT, thrombin time; INR, international normalized ratio; AG, anion gap; SOFA, sequential organ failure assessment; APACHE, acute physiology and chronic health evaluation; OR, odds ratio; CI, confidential interval.

### The results of the two-piecewise linear model

**Figure 1:**
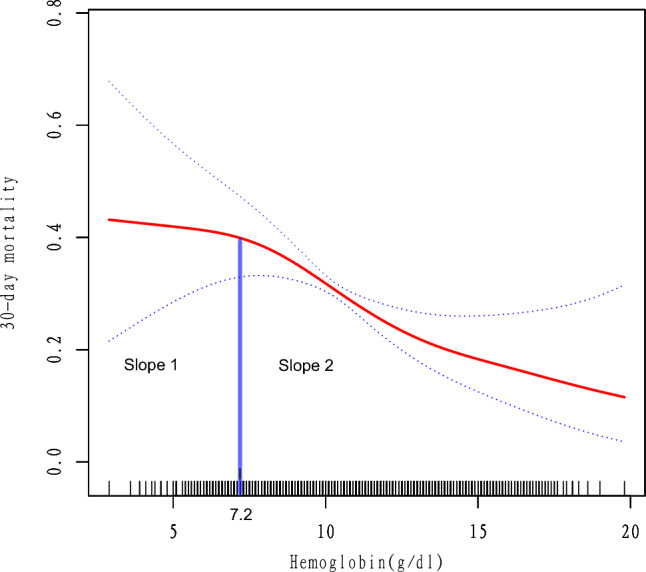
The smooth fitting curve indicated a non-linear relationship between hemoglobin level and 30-day mortality. Adjusted for age; gender; HR; SBP; DBP; RR; AG; ALT, AST, bicarbonate, INR, total bilirubin; total calcium; creatinine; chloride; hematocrit; lactate; PLT; PT; TT; RBC; urea nitrogen; WBC; sodium; renal disease; CAD; diabetes; hypertension; SOFA; APAHCEII. *Abbreviations:* ALT, alanine aminotransferase; AST, aspartate aminotransferase; CAD, coronary artery disease; SBP, systolic blood pressure; DBP, diastolic blood pressure; HR, heart rate; RR, respiratory rate; WBC, white blood cells; PLT, platelet; RBC, red blood cells; PT, prothrombin time; TT, thrombin time; INR, international normalized ratio; AG, anion gap; SOFA, sequential organ failure assessment; APACHE, acute physiology and chronic health evaluation.

In Table 4, standard linear model and two-piecewise linear model were applied to examine the better one for fitting the relationship between hemoglobin level and 30-day mortality. The P value for the log-likelihood ratio test was 0.011, which indicated that the two-piecewise linear model was the best. The smooth fitting curve (adjusted for all potential cofounders) for the relationship between hemoglobin level and 30-day mortality was performed in Fig. 1. The turning point was 7.2 g/dl. When hemoglobin level ≥ 7.2 g/dl (slope 2), the risk of 30-day mortality significantly decreased (OR = 0.85, 95%CI 0.78–0.93, *P = *0.0006). Compared slope 2(≥ 7.2 g/dl) to slope 1(< 7.2 g/dl), the OR was 0.68 (95%CI 0.51–0.93, *P = *0.0142). In Supplementary Table 2, the subgroup analyses were constructed and the results were comparatively stable in the subgroups.

**Table 4:** The results of the two-piecewise linear model.

	Number (%)	OR (95%CI), P
Fitting model by standard linear regression	6249 (100%)	0.87 (0.79, 0.95) 0.0020
Fitting model by two-piecewise linear regression		
The turning point of hemoglobin level (g/dl)		
< 7.2 (slope 1)	321 (5.14%)	1.24 (0.92, 1.68) 0.1596
≥ 7.2( slope 2)	5928 (94.86%)	0.85 (0.78, 0.93) 0.0006
Slope 2 to slope 1		0.68 (0.51, 0.93) 0.0142
Predicted at 7.2		− 0.69 (− 0.80, − 0.59)
P for the log-likelihood ratio test		0.011

Model adjusted for: age; gender; HR; SBP; DBP; RR; AG; ALT, AST, bicarbonate, INR, total bilirubin; total calcium; creatinine; chloride; hematocrit; lactate; PLT; PT; TT; RBC; urea nitrogen; WBC; sodium; renal disease; CAD; diabetes; hypertension; SOFA; APAHCEII.

ALT, alanine aminotransferase; AST, aspartate aminotransferase; CAD, coronary artery disease; SBP, systolic blood pressure; DBP, diastolic blood pressure; HR, heart rate; RR, respiratory rate; WBC, white blood cells; PLT, platelet; RBC, red blood cells; PT, prothrombin time; TT, thrombin time; INR, international normalized ratio; AG, anion gap; SOFA, sequential organ failure assessment; APACHE, acute physiology and chronic health evaluation; OR, odds ratio; CI, confidential interval.

### Kaplan–Meier analysis for cumulative hazard of 30-day mortality

**Figure 2:**
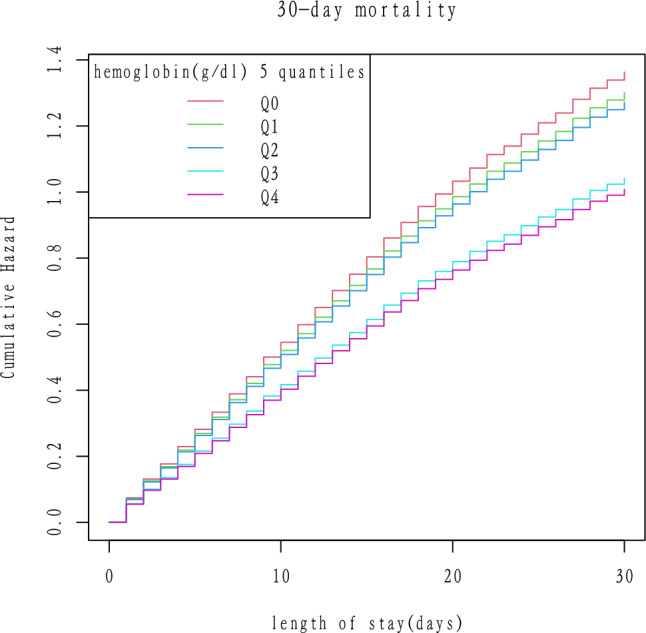
Kaplan–Meier analysis for cumulative hazard of 30-day mortality in sepsis.

Figure 2 illuminated Kaplan–Meier analysis for cumulative hazard of 30-day mortality. In Q0 group (≤ 8.4 g/dl), the cumulative hazard was the significantly highest (*P < *0.0001).

## Discussion

In the present study, several conclusions were made as follow: (1) The non-linear relationship between admission hemoglobin level and 30-day mortality in sepsis was found and the turning point was 7.2 g/dl; (2) With 1 g/dl increment in hemoglobin level, the OR of 30-day mortality decreased by 13% after adjusted for all potential cofounders (OR = 0.87, 95%CI 0.79- 0.95, *P = *0.0020); (3) Compared with the hemoglobin group* < *7.2 g/dl, the risk of 30-day mortality significantly decreased by 32% in the hemoglobin group ≥ 7.2 g/dl (OR = 0.68, 95%CI 0.51–0.93, *P = *0.0142). For septic patients, hemoglobin supplementation could be beneficial for those patients with hemoglobin level < 7.2 g/dl.

Hemoglobin level not only reflected the nutritional status but also was an indicator for the complications and outcomes^13–15^. A longitudinal analysis in old adults without anemia showed that higher hemoglobin levels were linked with better physical performance^16^. One observational study found that in patients with hip fracture, preoperative hemoglobin levels ≥ 10 g/dl lead to a 50% decreased risk of mortality compared to patients with hemoglobin levels < 10 g/dl (hazard ration(HR) = 0.50, 95% CI 0.25–0.99, *P = *0.048)^17^. Another research investigated the outcomes in patients with chronic kidney disease under cardiac surgery and indicated that with 1 g/dl decrement in hemoglobin, the values of OR in mortality, sepsis, cerebrovascular complication and postoperative hemodialysis were 1.38(95%CI 1.23–1.57; *P = *0.001), 1.31 (95%CI 1.14–1.49; *P = *0.001), 1.31 (95%CI 1.00–1.67; *P = *0.030), and 1.38 (95%CI 1.11–1.75; *P = *0.010), respectively^18^.

In sepsis, some studies also explored the effects of hemoglobin level on prognosis. For every increment in hemoglobin unit, the risk of worsened respiratory dysfunction in the following day decreased by 36% (OR = 0.64, 95%CI 0.53–0.77, *P < *0.001)^19^. One recent research in China concluded that septic patients with early hemoglobin levels ≤ 8 g/dl had significantly lower survival rates and was also identified as an indicator for predicting long-term outcomes^20^. In patients admitted in intensive medicine departments, hemoglobin level was strongly correlated with in-hospital mortality (OR = 0.83; 95%CI 0.74–0.92, *P = *0.0004)^21^, which partly was consistent with our results.

Several possible mechanisms can partly explain the close relationship between hemoglobin level and clinical outcomes in sepsis. First, in sepsis, oxygen demand in tissues usually increase significantly and lower hemoglobin can worsen the oxygen deficiency, leading to organ dysfunction and poorer outcomes^22^. Second, hemoglobin plays an important role in anti-inflammation by defending against bacteria and enhancing the function of leucocytes^23^. In addition, hemoglobin impacts on the metabolism and absorbs of antibiotics, while decreased level of hemoglobin significantly attenuate the anti-infection of antibiotics^24^.

Our research had some advantages. First, we analyzed the hemoglobin as a both continuous and categorical variable. Second, we applied a two-piecewise linear model to construct a threshold effect analysis on the relationship between hemoglobin level and mortality in sepsis. Third, subgroups analyses were performed, which could avoid the incidence of an occasionality in the statistical analysis as much as possible and improve the stability of the results.

Although the appropriate research methods were implemented, limitations and shortcomings should be mentioned. First, hemoglobin level could be affected by the fluid administration before drawing the blood sample. Second, due to the lack of some data, not all the factors which were associated with hemoglobin level were analyzed. Third, we didn’t analyze the effects of blood transfusion on prognosis in sepsis. Moreover, it was a retrospective study based on a U.S public database, so the limitations of applying the results should be considered. Most of the patients in MIMIC-IV database were Whites, it might be not suitable for applying the results to Asian countries. Considering the retrospective nature of the study, it is necessary to conduct studies such as randomized clinical trials or systematic reviews and meta-analyses for further validating our results. Fourth, due to the nature of retrospective study, we couldn’t identify the upper threshold hemoglobin value indicative of a favorable prognosis for sepsis patients in this study.

## Conclusion

The non-linear relationship between admission hemoglobin level and 30-day mortality in sepsis was found. Hemoglobin supplementation might be beneficial for septic patients with hemoglobin level < 7.2 g/dl.

### Supplementary Information

Supplementary Information 1.

Supplementary Information 2.

Supplementary Information 3.

## Data Availability

The data that support the findings of this study are available from the Massachusetts Institute of Technology (MIT) and Beth Israel Deaconess Medical Center (BIDMC) but restrictions apply to the availability of these data, which were used under license for the current study, and so are not publicly available. Data are however available from the authors upon reasonable request and with permission of the Massachusetts Institute of Technology (MIT) and Beth Israel Deaconess Medical Center (BIDMC). The datasets used and/or analyzed during the present study were availed by the corresponding author (Ning Ding) on reasonable request.
