# Supplementary figures and images for: Association between admission hemoglobin level and prognosis in sepsis patients based on a critical care database
Source: Sci Rep. 2024 Mar 3;14:5212. doi: 10.1038/s41598-024-55954-1 (PMC10909867; doi:10.1038/s41598-024-55954-1)

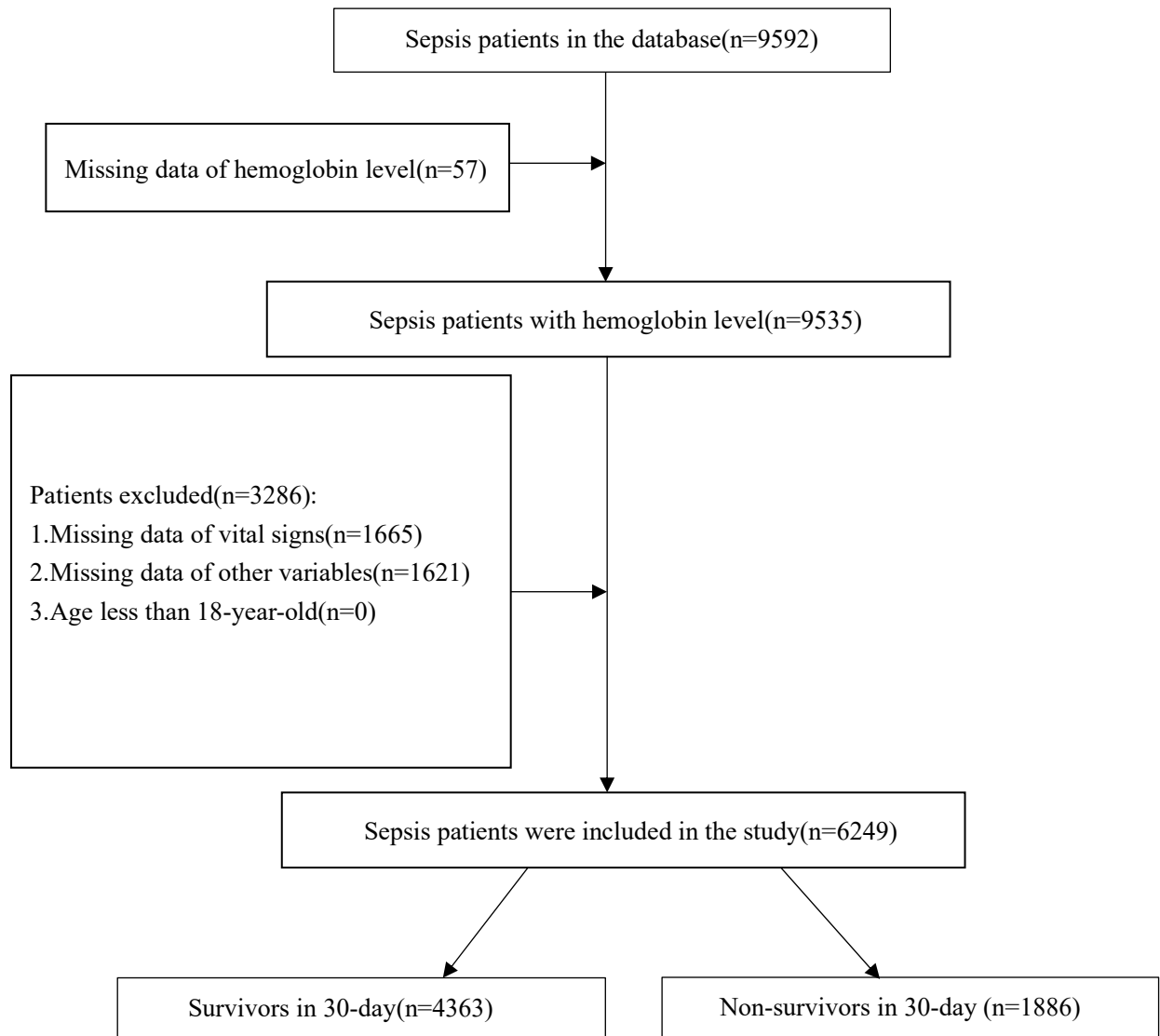

**Supplementary Figure 1: Study design and patients enrollment.**

Supplement: Supplementary file 1 — Supplementary Information 1. [file 41598_2024_55954_MOESM1_ESM.pdf]
