# Supplementary material for: Association between admission hemoglobin level and prognosis in sepsis patients based on a critical care database
Source: Sci Rep. 2024 Mar 3;14:5212. doi: 10.1038/s41598-024-55954-1 (PMC10909867; doi:10.1038/s41598-024-55954-1)
Supplement: Supplementary file 2 — Supplementary Information 2. [file 41598_2024_55954_MOESM2_ESM.docx]

**Supplementary Table 1 General characteristics of sepsis patients between five groups based on the quantiles of hemoglobin levels**

| Hemoglobin(g/dl) (quantiles) | | | | | | P-value |
| --- | --- | --- | --- | --- | --- | --- |
| Variables | Q0(≤8.4) | Q1(8.5-9.6) | Q2(9.7-10.8) | Q3(10.9-12.1) | Q4(≥12.2) |  |
| Number | 1189 | 1340 | 1190 | 1303 | 1263 |  |
| Age(years) | 64.00 (54.00-74.00) | 66.00 (55.00-77.00) | 67.00 (56.00-77.00) | 67.00 (57.00-78.00) | 66.00 (55.00-77.00) | <0.001 |
| Gender(n,%) |  |  |  |  |  | <0.001 |
| Male | 636 (53.49%) | 672 (51.53%) | 624 (52.44%) | 738 (56.64%) | 871 (68.96%) |  |
| Female | 553 (46.51%) | 632 (48.47%) | 566 (47.56%) | 565 (43.36%) | 392 (31.04%) |  |
| Comorbidities(n,%) |  |  |  |  |  |  |
| Hypertension | 158 (13.29%) | 262 (20.09%) | 263 (22.10%) | 302 (23.18%) | 317 (25.10%) | <0.001 |
| Diabetes | 35 (2.94%) | 58 (4.45%) | 38 (3.19%) | 46 (3.53%) | 26 (2.06%) | 0.015 |
| CAD | 59 (4.96%) | 130 (9.97%) | 123 (10.34%) | 131 (10.05%) | 127 (10.06%) | <0.001 |
| Renal disease | 39 (3.28%) | 77 (5.90%) | 70 (5.88%) | 60 (4.60%) | 25 (1.98%) | <0.001 |
| SBP(mmHg) | 109.00 (96.00-125.00) | 110.00 (97.00-128.00) | 110.00 (98.00-127.00) | 111.00 (98.00-130.00) | 112.00 (98.00-130.00) | 0.012 |
| DBP(mmHg) | 60.00 (52.00-72.00) | 60.00 (51.00-71.00) | 62.00 (53.00-74.00) | 64.00 (54.00-77.00) | 66.00 (56.00-78.00) | <0.001 |
| HR(beats/min) | 98.00 (84.00-113.00) | 95.00 (82.00-110.00) | 96.00 (82.00-112.00) | 97.00 (82.00-112.00) | 99.00 (84.00-114.00) | 0.002 |
| RR(beats/min) | 21.00 (17.00-26.00) | 20.00 (17.00-25.00) | 20.00 (17.00-24.00) | 21.00 (17.00-26.00) | 21.00 (17.00-26.00) | 0.048 |
| Hemoglobin(g/dl) | 7.60 (7.10-8.00) | 9.00 (8.70-9.30) | 10.20 (9.90-10.40) | 11.40 (11.10-11.70) | 13.30 (12.70-14.20) | <0.001 |
| RBC(*10^12^/l) | 2.58 (2.32-2.82) | 3.06 (2.83-3.29) | 3.40 (3.20-3.64) | 3.79 (3.56-4.04) | 4.36 (4.08-4.72) | <0.001 |
| Hematocrit(%) | 23.80 (22.10-25.30) | 28.20 (26.80-29.50) | 31.50 (30.20-32.80) | 35.10 (33.60-36.50) | 40.30 (38.30-43.40) | <0.001 |
| WBC(*10^9^/l) | 11.10 (6.50-17.20) | 12.30 (7.40-18.50) | 12.30 (7.82-18.48) | 13.20 (8.50-19.05) | 13.20 (8.65-18.90) | <0.001 |
| PLT(*10^9^/l) | 159.00 (84.00-266.00) | 192.00 (115.00-286.00) | 185.50 (123.00-269.00) | 188.00 (127.00-255.50) | 185.00 (129.00-248.00) | <0.001 |
| Creatinine(mg/dl) | 1.40 (0.90-2.50) | 1.50 (0.90-2.50) | 1.30 (0.90-2.30) | 1.30 (0.90-2.20) | 1.30 (0.90-2.00) | 0.003 |
| Urea nitrogen(mg/dl) | 32.00 (19.00-54.00) | 30.00 (20.00-50.00) | 29.00 (17.00-48.00) | 27.00 (18.00-43.00) | 26.00 (17.00-42.00) | <0.001 |
| AG(mmol/l) | 15.00 (12.00-19.00) | 15.00 (13.00-18.00) | 16.00 (13.00-19.00) | 16.00 (14.00-19.00) | 17.00 (14.00-20.00) | <0.001 |
| ALT(IU/L) | 25.00 (14.00-50.00) | 26.00 (15.00-53.25) | 29.00 (17.00-70.75) | 31.00 (17.00-71.00) | 36.00 (20.00-90.00) | <0.001 |
| AST(IU/L) | 39.00 (22.00-85.00) | 39.00 (23.00-83.00) | 45.00 (25.00-101.75) | 45.00 (25.00-104.00) | 52.00 (28.00-126.00) | <0.001 |
| Total bilirubin(mg/dl) | 0.70 (0.40-2.00) | 0.65 (0.40-1.60) | 0.70 (0.40-1.70) | 0.70 (0.40-1.70) | 0.90 (0.50-2.00) | <0.001 |
| Total calcium(mg/dl) | 7.90 (7.30-8.50) | 8.00 (7.50-8.50) | 8.00 (7.43-8.50) | 8.10 (7.50-8.70) | 8.20 (7.60-8.80) | <0.001 |
| Bicarbonate(mmol/l) | 21.00 (18.00-24.00) | 21.00 (18.00-25.00) | 21.00 (18.00-24.00) | 21.00 (18.00-25.00) | 21.00 (18.00-25.00) | 0.113 |
| INR | 1.50 (1.30-2.10) | 1.40 (1.20-1.90) | 1.40 (1.20-1.80) | 1.40 (1.20-1.80) | 1.40 (1.20-1.80) | <0.001 |
| PT(s) | 16.30 (13.90-22.10) | 15.50 (13.40-20.52) | 15.30 (13.30-19.78) | 15.10 (13.20-19.20) | 14.90 (12.80-19.30) | <0.001 |
| TT(s) | 34.20 (29.40-42.60) | 33.15 (29.10-41.12) | 32.80 (28.70-40.10) | 32.20 (28.10-39.70) | 32.70 (28.30-39.85) | <0.001 |
| Lactate(mmol/l) | 1.80 (1.20-2.90) | 1.80 (1.30-2.80) | 2.00 (1.40-3.10) | 2.10 (1.50-3.20) | 2.40 (1.60-3.60) | <0.001 |
| Chloride(mmol/l) | 103.00 (98.00-108.00) | 103.00 (98.00-108.00) | 103.00 (98.00-108.00) | 103.00 (98.00-108.00) | 103.00 (98.00-107.00) | 0.580 |
| Sodium(mmol/l) | 137.00 (134.00-141.00) | 137.00 (134.00-141.00) | 138.00 (134.00-141.00) | 138.00 (134.00-141.00) | 138.00 (135.00-141.00) | 0.006 |
| SOFA | 3.00 (2.00-5.00) | 3.00 (2.00-5.00) | 3.00 (2.00-4.00) | 3.00 (2.00-4.00) | 2.00 (2.00-4.00) | <0.001 |
| APACHEII | 13.00 (11.00-16.00) | 13.00 (10.00-16.00) | 11.00 (9.00-14.00) | 11.00 (8.00-14.00) | 11.00 (9.00-14.00) | <0.001 |
| **Outcomes** |  |  |  |  |  |  |
| LOS in ICU(days) | 4.55 (2.13-10.31) | 4.64 (2.19-9.73) | 4.55 (2.16-10.51) | 4.80 (2.13-10.73) | 5.27 (2.21-11.83) | 0.261 |
| LOS in hospital(days) | 12.28 (6.76-22.43) | 11.07 (6.65-20.29) | 11.59 (6.49-19.58) | 11.06 (6.00-20.76) | 11.71 (5.93-21.45) | 0.115 |
| 30-day mortality(n,%) | 407 (34.23%) | 408 (31.29%) | 362 (30.42%) | 357 (27.40%) | 352 (27.87%) | 0.001 |

**Abbreviations:** ALT=alanine aminotransferase, AST= aspartate aminotransferase, CAD= coronary artery disease, SBP=systolic blood pressure, DBP= diastolic blood pressure, HR= heart rate, RR=respiratory rate, WBC=white blood cells, PLT=platelet, RBC=red blood cells, PT= prothrombin time, TT=thrombin time, INR=international normalized ratio, AG=anion gap, SOFA=sequential organ failure assessment, APACHE=acute physiology and chronic health evaluation, LOS=length of stay, ICU=intensive care unit, IQR=interquartile ranges.
