# Supplementary material for: Association between admission hemoglobin level and prognosis in sepsis patients based on a critical care database
Source: Sci Rep. 2024 Mar 3;14:5212. doi: 10.1038/s41598-024-55954-1 (PMC10909867; doi:10.1038/s41598-024-55954-1)
Supplement: Supplementary file 3 — Supplementary Information 3. [file 41598_2024_55954_MOESM3_ESM.docx]

**Supplementary Table 2: Subgroup analyses**

| Variables | Number | OR(95%CI) | P for interaction |
| --- | --- | --- | --- |
| Gender |  |  | 0.0139 |
| Male | 3541 | 0.94 (0.91, 0.97) 0.0001 |  |
| Female | 2708 | 1.00 (0.96, 1.05) 0.8870 |  |
| Age, years (tertiles) |  |  | 0.1372 |
| ≤58 | 1962 | 0.92 (0.88, 0.96) 0.0006 |  |
| 59-73 | 2131 | 0.97 (0.93, 1.02) 0.2126 |  |
| ≥74 | 2156 | 0.97 (0.93, 1.02) 0.2317 |  |
| Hypertension |  |  | 0.7045 |
| No | 4947 | 0.96 (0.93, 0.99) 0.0043 |  |
| Yes | 1302 | 0.97 (0.92, 1.03) 0.3221 |  |
| Diabetes |  |  | 0.0308 |
| No | 6046 | 0.96 (0.94, 0.99) 0.0056 |  |
| Yes | 203 | 0.79 (0.65, 0.95) 0.0137 |  |
| CAD |  |  | 0.8012 |
| No | 5679 | 0.96 (0.94, 0.99) 0.0031 |  |
| Yes | 570 | 0.95 (0.87, 1.04) 0.2536 |  |
| Renal disease |  |  | 0.2593 |
| No | 5978 | 0.96 (0.94, 0.98) 0.0017 |  |
| Yes | 271 | 1.04 (0.91, 1.20) 0.5587 |  |
| HR, beats/min (tertiles) |  |  | 0.9450 |
| ≤86 | 2065 | 0.96 (0.92, 1.01) 0.1177 |  |
| 87-107 | 2089 | 0.96 (0.92, 1.01) 0.1082 |  |
| ≥108 | 2095 | 0.96 (0.92, 1.00) 0.0300 |  |
| DBP, mmHg (tertiles) |  |  | 0.0689 |
| ≤55 | 1928 | 1.00 (0.95, 1.04) 0.9580 |  |
| 56-70 | 2200 | 0.97 (0.93, 1.02) 0.1988 |  |
| ≥71 | 2121 | 0.93 (0.89, 0.97) 0.0006 |  |
| SBP, mmHg(tertiles) |  |  | 0.1016 |
| ≤100 | 2068 | 0.98 (0.94, 1.03) 0.4435 |  |
| 101-121 | 2042 | 0.98 (0.94, 1.02) 0.3740 |  |
| ≥122 | 2139 | 0.93 (0.89, 0.97) 0.0006 |  |
| RR, beats/min (tertiles) |  |  | 0.0858 |
| ≤17 | 1775 | 0.95 (0.90, 1.00) 0.0307 |  |
| 18-24 | 2386 | 1.00 (0.96, 1.04) 0.8495 |  |
| ≥25 | 2088 | 0.93 (0.90, 0.97) 0.0015 |  |
| WBC, *10^9^/l (tertiles) |  |  | 0.9051 |
| ≤9.1 | 2071 | 0.97 (0.93, 1.01) 0.1244 |  |
| 9.2-16.2 | 2081 | 0.97 (0.93, 1.01) 0.1237 |  |
| ≥16.3 | 2097 | 0.95 (0.91, 1.00) 0.0362 |  |
| AG, mmol/l (tertiles) |  |  | 0.8473 |
| ≤13 | 1740 | 0.96 (0.91, 1.02) 0.1658 |  |
| 14-18 | 2296 | 0.95 (0.91, 0.99) 0.0170 |  |
| ≥19 | 2213 | 0.95 (0.91, 0.98) 0.0047 |  |
| RBC, *10^12^/l (tertiles) |  |  | 0.8366 |
| ≤3.10 | 2078 | 1.00 (0.93, 1.08) 0.9902 |  |
| 3.11-3.79 | 2078 | 1.02 (0.94, 1.11) 0.6764 |  |
| ≥3.80 | 2093 | 1.03 (0.97, 1.09) 0.3151 |  |
| Total bilirubin, mg/dl (tertiles) |  |  | 0.2858 |
| ≤0.4 | 1814 | 0.99 (0.94, 1.05) 0.7014 |  |
| 0.5-1.3 | 2348 | 0.96 (0.92, 1.00) 0.0296 |  |
| ≥1.4 | 2087 | 0.94 (0.90, 0.97) 0.0011 |  |
| Total calcium, mg/dl(tertiles) |  |  | 0.4253 |
| ≤7.6 | 1981 | 0.95 (0.91, 0.99) 0.0267 |  |
| 7.7-8.4 | 2004 | 0.94 (0.90, 0.99) 0.0152 |  |
| ≥8.5 | 2264 | 0.98 (0.94, 1.02) 0.3053 |  |
| ALT, IU/L (tertiles) |  |  | 0.8483 |
| ≤19 | 2041 | 0.96 (0.91, 1.00) 0.0734 |  |
| 20-48 | 2100 | 0.95 (0.91, 0.99) 0.0127 |  |
| ≥49 | 2108 | 0.96 (0.92, 1.00) 0.0689 |  |
| AST, IU/L(tertiles) |  |  | 0.8992 |
| ≤28 | 2028 | 0.96 (0.91, 1.00) 0.0700 |  |
| 29-72 | 2111 | 0.95 (0.90, 0.99) 0.0169 |  |
| ≥73 | 2110 | 0.94 (0.91, 0.98) 0.0027 |  |
| Chloride, mmol/l(tertiles) |  |  | 0.9449 |
| ≤99 | 1933 | 0.96 (0.92, 1.00) 0.0391 |  |
| 100-106 | 2119 | 0.96 (0.92, 1.00) 0.0516 |  |
| ≥107 | 2197 | 0.97 (0.92, 1.01) 0.1233 |  |
| Creatinine, mg/dl(tertiles) |  |  | 0.2811 |
| ≤0.9 | 1794 | 1.00 (0.95, 1.05) 0.8845 |  |
| 1.0-1.9 | 2369 | 0.95 (0.91, 0.98) 0.0059 |  |
| ≥2.0 | 2086 | 0.97 (0.93, 1.01) 0.1280 |  |
| Hematocrit, %(tertiles) |  |  | 0.0455 |
| ≤28.6 | 2064 | 0.95 (0.87, 1.03) 0.2120 |  |
| 28.7-34.6 | 2097 | 0.83 (0.74, 0.93) 0.0009 |  |
| ≥34.7 | 2088 | 0.97 (0.91, 1.04) 0.4321 |  |
| INR (tertiles) |  |  | 0.6116 |
| ≤1.2 | 1936 | 0.96 (0.92, 1.01) 0.1330 |  |
| 1.3-1.6 | 1937 | 0.99 (0.95, 1.04) 0.7432 |  |
| ≥1.7 | 2376 | 0.97 (0.93, 1.00) 0.0801 |  |
| Lactate, mmol/l(tertiles) |  |  | 0.3963 |
| ≤1.5 | 2041 | 0.94 (0.89, 1.00) 0.0366 |  |
| 1.6-2.7 | 2116 | 0.95 (0.91, 1.00) 0.0379 |  |
| ≥2.8 | 2092 | 0.92 (0.88, 0.95) <0.0001 |  |
| PLT, *10^9^/l(tertiles) |  |  | 0.1613 |
| ≤138 | 2059 | 0.97 (0.93, 1.01) 0.1326 |  |
| 139-233 | 2096 | 0.94 (0.90, 0.98) 0.0075 |  |
| ≥234 | 2094 | 1.00 (0.96, 1.05) 0.9942 |  |
| PT, s(tertiles) |  |  | 0.1288 |
| ≤13.8 | 2016 | 0.96 (0.91, 1.00) 0.0616 |  |
| 13.9-17.9 | 2121 | 1.01 (0.97, 1.06) 0.6298 |  |
| ≥18.0 | 2112 | 0.96 (0.92, 1.00) 0.0283 |  |
| TT, s (tertiles) |  |  | 0.6018 |
| ≤30.0 | 2062 | 0.97 (0.92, 1.01) 0.1486 |  |
| 30.1-37.5 | 2099 | 0.99 (0.94, 1.03) 0.5347 |  |
| ≥37.6 | 2088 | 0.96 (0.92, 1.00) 0.0286 |  |
| Sodium, mmol/l (tertiles) |  |  | 0.7763 |
| ≤134 | 1702 | 0.97 (0.93, 1.02) 0.2543 |  |
| 135-140 | 2332 | 0.95 (0.91, 0.99) 0.0235 |  |
| ≥141 | 2215 | 0.97 (0.93, 1.01) 0.1520 |  |
| Urea nitrogen, mg/dl (tertiles) |  |  | 0.7408 |
| ≤20 | 1980 | 0.98 (0.93, 1.03) 0.3813 |  |
| 21-40 | 2184 | 0.96 (0.92, 1.00) 0.0553 |  |
| ≥41 | 2085 | 0.98 (0.94, 1.02) 0.3685 |  |
| Bicarbonate, mmol/l (tertiles) |  |  | 0.0408 |
| ≤18 | 1800 | 1.01 (0.97, 1.05) 0.7059 |  |
| 19-23 | 1943 | 0.94 (0.90, 0.98) 0.0089 |  |
| ≥24 | 2506 | 0.94 (0.90, 0.98) 0.0046 |  |
| SOFA |  |  | 0.8333 |
| ≤2 | 2852 | 0.97 (0.93, 1.01) 0.1170 |  |
| 3-4 | 1831 | 0.97 (0.93, 1.02) 0.2682 |  |
| ≥5 | 1566 | 0.99 (0.95, 1.03) 0.6437 |  |
| APACHEII |  |  | 0.0387 |
| ≤10 | 1699 | 0.92 (0.87, 0.98) 0.0114 |  |
| 11-13 | 2206 | 0.98 (0.94, 1.02) 0.3468 |  |
| ≥14 | 2344 | 1.01 (0.98, 1.05) 0.4901 |  |

**Abbreviations:** ALT=alanine aminotransferase, AST= aspartate aminotransferase, CAD= coronary artery disease, SBP=systolic blood pressure, DBP= diastolic blood pressure, HR= heart rate, RR=respiratory rate, WBC=white blood cells, PLT=platelet, RBC=red blood cells, PT= prothrombin time, TT=thrombin time, INR=international normalized ratio, AG=anion gap, SOFA=sequential organ failure assessment, APACHE=acute physiology and chronic health evaluation, OR=odds ratio, CI=confidential interval.
